# A cluster‐randomized controlled trial of a combination HIV risk reduction and microfinance intervention for female sex workers who use drugs in Kazakhstan

**DOI:** 10.1002/jia2.25682

**Published:** 2021-05-05

**Authors:** Nabila El‐Bassel, Tara McCrimmon, Gaukhar Mergenova, Mingway Chang, Assel Terlikbayeva, Sholpan Primbetova, Azamat Kuskulov, Bauyrzhan Baiserkin, Alfiya Denebayeva, Kulpan Kurmetova, Susan S. Witte

**Affiliations:** ^1^ Global Health Research Center of Central Asia Columbia University School of Social Work New York NY USA; ^2^ Global Health Research Central Asia Almaty Kazakhstan; ^3^ Kazakh Scientific Center for Dermatology and Infectious Diseases Almaty Kazakhstan; ^4^ Almaty City Center of the Prevention and Control of AIDS Almaty Kazakhstan; ^5^ Temirtau Branch Karaganda Oblast Center for the Prevention and Control of AIDS Temirtau Kazakhstan

**Keywords:** structural interventions, sex workers, drug use, HIV prevention, clinical trials

## Abstract

**Introduction:**

Female sex workers (FSW) who use drugs are a key population at risk of HIV in Kazakhstan, and face multiple structural barriers to HIV prevention. More research is needed on the role of structural interventions such as microfinance (MF) in reducing HIV risk. This paper describes the results of a cluster‐randomized controlled trial to test the efficacy of a combination HIVRR + MF intervention in reducing biologically confirmed STIs and HIV risk behaviours.

**Methods:**

This study took place from May 2015 to October 2018 in two cities in Kazakhstan. We screened 763 participants for eligibility and enrolled 354 FSW who use drugs. Participants were randomized in cohorts to receive either a four‐session HIVRR intervention, or that same intervention plus 30 additional sessions of financial literacy training, vocational training and asset‐building through a matched‐savings programme. Repeated behavioural and biological assessments were conducted at baseline, 3‐, 6‐ and 12‐months post‐intervention. Biological and behavioural primary outcomes included HIV/STI incidence, sexual risk behaviours and drug use risk behaviours, evaluated over the 12‐month period.

**Results:**

Over the 12‐month follow‐up period, few differences in study outcomes were noted between arms. There was only one newly‐detected HIV case, and study arms did not significantly differ on any STI incidence. At post‐intervention assessments compared to baseline, both HIVRR and HIVRR + MF participants significantly reduced sexual and drug use risk behaviours, and showed improvements in financial outcomes, condom use attitudes and self‐efficacy, social support, and access to medical care. In addition, HIVRR + MF participants showed a 72% greater reduction in the number of unprotected sex acts with paying partners at the six‐month assessment (IRR = IRR = 0.28, 95% CI = 0.08, 0.92), and a 10% greater reduction in the proportion of income from sex work at the three‐month assessment (b = −0.10, 95% CI = −0.17, −0.02) than HIVRR participants did. HIVRR + MF participants also showed significantly improved performance on financial self‐efficacy compared to HIVRR over the 12‐month follow‐up period.

**Conclusions:**

Compared to a combination HIVRR + MF intervention, a robust HIVRR intervention alone may be sufficient to reduce sexual and drug risk behaviours among FSW who use drugs. There may be structural limitations to the promise of microfinance for HIV risk reduction among this population.

## 1 INTRODUCTION

Female sex workers (FSW) are a key population at high risk for HIV and other sexually transmitted infections (STIs). In low and middle‐income countries, estimates put their odds of HIV infection at 13.5 times those of all women of reproductive age [1]. While injection drug use has historically driven the HIV epidemic in Central Asia, there is growing attention to the role of FSW [2]. Kazakhstan has an estimated 21,450 FSW, 1.4% of whom are HIV positive [3]. An estimated 2.3% of Kazakhstan’s FSW inject drugs; there are no estimates of non‐injection drug use among FSW [3]. There is a lack of structural HIV prevention interventions among women who use drugs, part of a larger trend of women’s underrepresentation in substance use research [4, 5]. Some evidence suggests that HIV burden may be even higher among FSW who use injection or non‐injection drugs [2]. While often considered distinct populations, FSW who are also injection or non‐injection drug users face overlapping physical, social, economic and policy risk environments that may limit their ability to negotiate safe sex, practice safe injection, protect themselves from violence from clients, managers, or intimate partners, or seek necessary medical or social services [6, 7]. Substance use may drive entry into sex work, and may exacerbate risks faced by FSW, reducing their capacity to insist on client condom use and increasing their experience of violence, including by police [7]. These structural barriers also limit access to treatment and care.

Structural interventions such as microfinance have the potential to address the economic risk environment faced by FSW who use drugs. The promise of microfinance for HIV risk reduction has received much interest, though few empirical results [8]. Microfinance interventions for HIVRR can take many forms – reviews have highlighted a range of programmes, including financial‐literacy education, vocational training, conditional or unconditional cash transfers, formal or informal microcredit or lending, small‐business development and asset‐building through savings programmes [9, 10, 11]. Few microfinance studies have been conducted among FSW, and only one among FSW who use drugs. The results suggest that among FSW, receiving a microfinance intervention may be associated with a reduced number of paying partners [12, 13, 14, 15], increased condom use [12, 14, 15] and alternative income [13, 15]. They suggest that providing women with increased economic opportunities decreases their reliance on sex work and high‐risk unprotected sex for income. However, existing studies insufficiently address empirical concerns, as one lacked a controlled design and none included biological outcome measures.

The Nova study addresses these gaps by rigorous evaluation of a combination HIV Risk Reduction (HIVRR) and microfinance (MF) intervention among FSW who used drugs in Kazakhstan. We hypothesized that compared to those who received the HIVRR intervention alone, participants who received a combination HIVRR + MF intervention would see (1) lower cumulative incidence of biologically confirmed STIs (gonorrhoea, trichomoniasis, chlamydia), (2) lower rate of new HIV cases, (3) greater decrease in the number of unprotected vaginal and anal sexual acts and a greater increase in the proportion of protected sexual acts with both intimate and paying partners, (4) reduction in the proportion of unsafe injection acts for those participants who inject drugs, and (5) lower proportion of monthly income from sex work.

## 2 METHODS

This study utilized a cluster‐randomized controlled trial (cRCT) design to compare participants receiving HIVRR alone and participants receiving HIVRR + MF. Recruitment and enrolment began in May 2015, and follow‐up data collection ended in October 2018. All procedures received approval from the Institutional Review Board (IRB) at Columbia University and the Ethics Committee of the Kazakhstan School of Public Health.

### 2.1 Recruitment of participants

The study took place in Almaty and Temirtau, two cities in Kazakhstan. Information on these cities is available elsewhere [16]. We recruited participants from NGOs, medical and social service organizations, and peer referrals. Research assistants distributed informational brochures and conducted outreach to provide study information to potential participants.

### 2.2 Screening for eligibility

Research assistants administered a brief computer‐based eligibility screening. Participants were eligible if they reported: (a) being over 18 years old; (b) illicit drug use within the past 12 months; (c) having exchanged sex for money, goods, drugs, or services within the past 90 days and (d) at least one incidence of unprotected sex (with either a paying or non‐paying partner) within the past 90 days. Participants were ineligible if they (a) could not communicate in Russian; (b) intended to move from the study site within the next year or (c) were determined to have cognitive impairment that would affect their ability to provide consent or participate fully. Participants received $1 for screening completion.

### 2.3 Study procedures

#### 2.3.1 Randomization

Eligible participants completed an informed consent process at a Nova field office, followed by a baseline assessment (see below). Within two weeks of baseline, participants were enrolled into 53 cohorts of six to eight participants. We randomized each cohort to either the treatment (HIVRR + MF) or control (HIVRR only) arm of the study through the use of a random number generator, conducted by the study biostatistician. Neither participants nor study staff were blind to study arm assignment.

#### 2.3.2 Intervention

Detailed information on intervention components, intervention selection, and adaptation is provided elsewhere [17]. Both arms received four HIVRR sessions. Participants assigned to the combination HIVRR + MF treatment arm received 30 additional sessions, including (1) six financial literacy training sessions, (2) 24 vocational training sessions in hairdressing, sewing, or manicurist professions and (3) a matched‐savings programme incentivizing them to accumulate assets for small‐business development or job/vocational training. Participants received small financial incentives ($12 per session attended), as well as small safe‐sex kits of condoms and lubricant. Sessions included brief safety check‐ins assessing whether participants faced risks of violence related or unrelated to study participation. Facilitators brought all expressed referral needs to the research team, who worked to link participants to requested services.

##### HIV risk reduction

Participants in both study arms received four 2‐hour HIVRR sessions, delivered by trained facilitators over two weeks. HIVRR focused on sexual and drug use risk reduction, and aimed to increase communication, problem‐solving skills, and self‐efficacy related to safe‐sex behaviours and drug use.

##### Financial literacy training

Only the HIVRR + MF arm received six 2‐hour FLT sessions, delivered by trained facilitators over two weeks. FLT focused on facilitating access to banking services, household budgeting, short and long‐term savings, and debt management.

Intervention facilitators for both components received weekly supervision from the research team. Audio recording and review by the research team were used for ongoing training.

##### Vocational training

After completing FLT, the HIVRR + MF arm received 24 sessions (two months) of Vocational Training (VT) in either hairdressing, sewing, or manicurist education, conducted by community‐based institutes or individuals.

##### Matched savings

HIVRR + MF arm participants received matched savings during FLT and VT. For every dollar of incentive payment ($12 per session) deposited in a personal bank account, the project deposited an equal amount in a separate, project‐controlled account under the participant’s name. Participants who saved 100% of their incentive would receive a matching $360 for a total of $720. For up to six months following the last VT session, participants could use this matched amount to purchase items related to their chosen vocation or to pay for continuing education in their vocation.

#### 2.3.3 Baseline and follow‐up assessments

**Figure 1:**
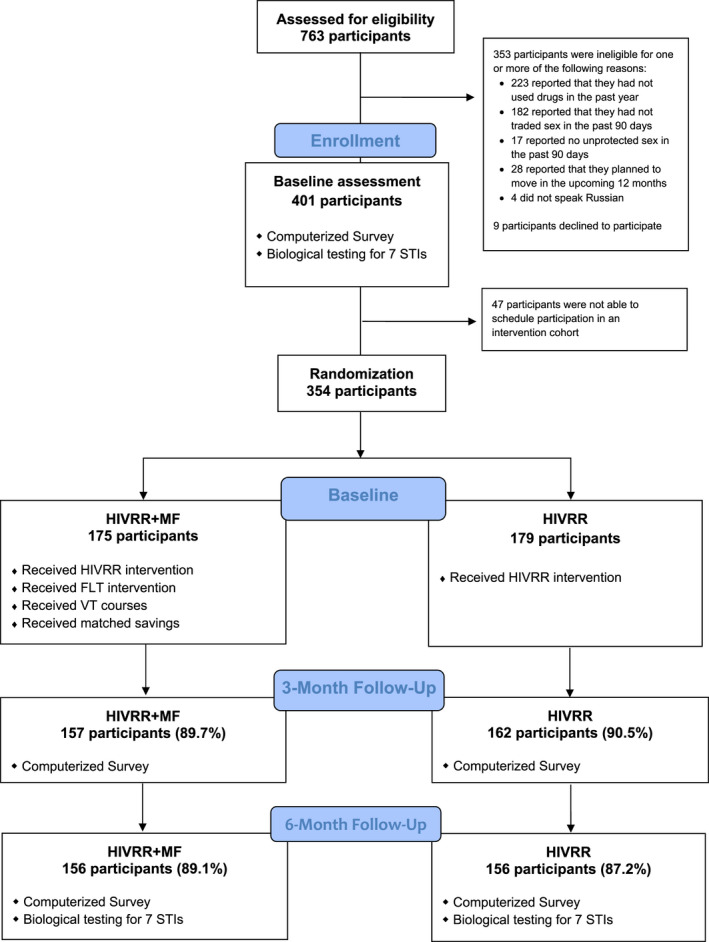
CONSORT flow diagram.

Assessments took place at pre‐intervention baseline (prior to cohort assignment and randomization), then at three, six, and twelve months following intervention completion. Follow‐up assessments allocated equivalent time for both arms, for example the 2.5 months for the intervention sessions (see Figure 1). Each assessment consisted of a behavioural questionnaire conducted using computer‐assisted self‐interviewing (CASI) and baseline, 6, and 12 months included biological assays for HIV and three STIs, conducted by a clinical coordinator in each field office. Participants received the equivalent of US $10, $9, $11, and $16 for completion of baseline, 3‐month, 6‐month, and 12‐month follow‐up assessments respectively.

##### Outcomes

Aligned with our study hypotheses, our primary biological and behavioural outcomes included:

1. Incidence of biologically confirmed STIs (gonorrhoea, chlamydia, trichomoniasis).
2. Rate of new HIV cases.
3. Number and proportion of unprotected vaginal and anal sex acts with both regular and paying partners.
4. Whether participant used injection drugs.
5. Whether participant performed unsafe injection.
6. Proportion of monthly income received from sex work.

Secondary outcomes included: the number of paying/non‐paying partners, condom use self‐efficacy, condom use attitudes, financial self‐efficacy, financial safety, perceived social support, and self‐reported access to care.

##### Measures

A complete list of measures is available elsewhere [16].

*Sociodemographic* information collected included age, ethnicity, marital status, education, and income.

To assess both *sexual risk behaviours* and *drug use risk behaviours*, we utilized a modified Risk Behaviour Assessment (RBA) [18], including data on type and frequency of drugs used, number of sexual partners (paying and intimate) in the past 90 days, and sex acts and condom use with these partners. We used Likert‐scale measures (detailed in Table 3) to measure *attitudes towards condom use, condom use self‐efficacy, social support* [19], and *access to care* [18].

For *financial outcomes* participants reported their total income and the proportion from sex work. Participants also completed measures of *financial self‐efficacy* and *financial safety* [20, 21].

For *b* *iotesting* we selected three STIs (gonorrhoea, chlamydia, trichomoniasis) as proxies of sexual risk behaviour, based on their high prevalence and incidence in FSW populations and the availability of definitive treatment with a single‐dose medication. Clinical coordinators collected vaginal swab samples to assess these STIs, using the AmpliSens N.gonorrhoeae/C.trachomatis/T.vaginalis‐MULTIPRIME‐FRT PCR assay (sensitivity = 95.1%, specificity = 99.4%). We assessed HIV through blood samples collected from participants using the Alere Determine™ HIV‐1/2 Ag/Ab Combo rapid test system (sensitivity = 100%, specificity = 98.80%). Participants who tested positive for HIV or STIs immediately received referrals and treatment.

#### 2.3.4 Statistical analysis

Power analyses suggested that a sample size of 350, randomizing in equal proportions to each arm (175 participants each), would achieve 80% power to detect STI incidence relative risks equal to 0.25 comparing women randomized to HIVRR + MF to HIVRR alone. These power analyses took into account baseline prevalence estimates from our prior work in similar populations [22], modest intra‐class correlation coefficient equal to 0.01 for clustering effects, and 20% attrition over the follow‐up period. The details can be found in the study protocol paper [16].

We employed statistical methods consistent with the intention‐to‐treat approach. For biological outcomes, we calculated person‐year incidence rates for HIV and STIs by arms. We defined “any STI” as any new infection of gonorrhoea, chlamydia or trichomoniasis detected at six or 12‐month follow‐up. Poisson regressions with robust variance were used to assess differences between arms. For self‐report outcomes, we employed mixed‐effects models to estimate effects of the change from baseline to each follow‐up for both arms and to test differences of change between two arms. Random effect parameters for repeated measures over time were included to handle non‐independence in observations. Each mixed model also included arm, follow‐up time, the interaction between arm and follow‐up time, and covariate adjustments for age, education, marital status, homelessness, food insecurity, and research sites. We used mixed‐effects Poisson regression to estimate effects on the number of unprotected vaginal intercourse acts and the number of sex partners. We reported estimates of effects as incident rate ratios (IRR) and corresponding 95% confidence intervals (CIs). We used mixed‐effects logistic regression to estimate effects on injection drug use, and reported odds ratios (OR) and 95% CIs. We employed mixed‐effects linear regression for financial outcomes, condom use attitudes, social support, and access to care. For these effect estimates, we report regression coefficients (b) and 95% CIs. All statistical analyses were performed using Stata (version 15.1).

## 3 RESULTS

We screened 763 individuals of whom 401 met eligibility criteria and completed the baseline assessment. Of these, 354 were enrolled and randomized into either the HIVRR + MF arm (n = 175) or the HIVRR arm (n = 179). There were no significant differences between study arms with respect to overall retention rates (HIVRR + MF = 92.67% vs. HIVRR = 92.7; retention rates at each follow‐up are provided in Figure 1). Both arms had high HIVRR attendance (average of 3.5 of 4 sessions). HIVRR + MF participants had an average session attendance of 4.9 out of 6 and 19.6 out of 24, for FLT and VT courses respectively. Over 75% (n = 133) of HIVRR + MF participants were eligible for the matched savings, but only 86 (49.1%) deposited money. The average deposit was US $149. Challenges to intervention uptake, as well as barriers to matched‐savings adherence, have been presented elsewhere [17]. Investigators identified no safety concerns related to intervention participation. Table 1 describes sociodemographic characteristics and criminal justice involvement at baseline assessment for 354 participants by intervention assignment. Sociodemographic characteristics and justice involvement variables did not differ significantly between the arms.

**Table 1:** Descriptive statistics of the sample reported at the baseline assessment

	Total (N = 354)^a^	HIVRR (n = 179)^a^	HIVRR + MF (n = 175)
Age, mean (SD)	34.0 (8.4)	34.0 (8.4)	34.1 (8.4)
Almaty site	222 (62.9%)	109 (61.2%)	113 (64.6%)
Russian	238 (67.4%)	119 (66.9%)	119 (68.0%)
Completed high school and above	239 (67.7%)	120 (67.4%)	119 (68.0%)
Single, never married	110 (31.2%)	55 (30.9%)	55 (31.4%)
Married or common law marriage	96 (27.2%)	48 (27.0%)	48 (27.4%)
Homeless in the past 90 day	204 (57.8%)	109 (61.2%)	95 (54.3%)
Not enough money to buy food in the past 90 days	319 (90.4%)	162 (91.0%)	157 (89.7%)
Years in sex work, mean (SD)	9.8 (7.3)	10.2 (7.2)	9.3 (7.3)
Ever been arrested or detained	275 (77.9%)	138 (77.5%)	137 (78.3%)
Ever been charged	178 (50.4%)	87 (48.9%)	91 (52%)
Ever been in jail or prison	111 (31.4%)	55 (30.9%)	56 (32.0%)
Been arrested or detained in the past 90 days	100 (28.3%)	53 (29.8%)	47 (26.9%)
Been charged in the past 90 days	24 (6.8%)	12 (6.7%)	12 (6.9%)
Been in jail or prison in the past 90 days	11 (3.1%)	5 (2.8%)	6 (3.4%)

HIVRR, HIV risk reduction; HIVRR + MF, HIV risk reduction and microfinance.

^a^ 1 Missing case.

Table 2 reports per 100 person‐year incidence rates for HIV and STIs by arm. There was only one newly detected HIV case with 153.3 person‐years in the HIVRR arm (i.e. 0.7 per 100 person‐years) over the one‐year follow‐up period. There were 41 newly detected STI cases with 130.3 person‐years in the HIVRR arm (i.e. 31.5 per 100 person‐years) and 39 new STI cases with 130.3 person‐years in the HIVRR + MF arm (i.e. 29.9 per 100 person‐years). The study arms did not significantly differ on HIV, gonorrhoea, chlamydia, trichomoniasis, and any STI incidence.

**Table 2:** Per 100 person‐year STI incidence rates by conditions

	Arm	Baseline (#)	New incidence at each follow‐up (#)		Person year	# Positive over one year	Per 100 persons per year incidence rates
			6 Month	12 Month			
HIV	HIVRR	41	1	0	153.3	1	0.7 [0.1, 4.6]
	HIVRR + MF	40	0	0	153.3	0	0 [–, –]
Gonorrhoea	HIVRR	5	2	1	153	3	2.0 [0.6, 6.1]
	HIVRR + MF	3	4	0	150.5	4	2.7 [1.0, 7.1]
Chlamydia	HIVRR	21	9	9	147	15	10.2 [6.2, 16.9]
	HIVRR + MF	15	10	7	144.8	16	11.1 [6.8, 18.0]
Trichomoniasis	HIVRR	34	21	16	136.8	29	21.2 [14.7, 30.5]
	HIVRR + MF	31	16	15	139.5	23	16.5 [11.0, 24.8]
Any STI	HIVRR	53	29	23	130.3	41	31.5 [23.2, 42.8]
	HIVRR + MF	44	27	21	130.3	39	29.9 [21.9, 41.0]

HIVRR, HIV risk reduction; HIVRR + MF, HIV risk reduction and microfinance.

Table 3 presents descriptive statistics of both primary and secondary behavioural outcome measures reported at the baseline, 3, 6, and 12‐month follow‐up assessments by intervention assignment. HIVRR + MF participants reported a significantly lower average proportion of income from sex work (0.15 vs. 0.23), greater average scores on financial safety (17.1 vs. 16.4) and condom use attitudes (22.3 vs. 20.9) than HIVRR participants at the 6‐month assessment. HIVRR + MF participants also reported greater average scores on perceived social support at three months and 12 months (57.8 vs. 49.9 and 57.6 vs. 51.8 respectively) and on access to care at all assessments (17.8 vs. 16.4 at baseline, 19.1 vs. 17.7 at three months, 19.5 vs. 18.0 at six months and 20.5 vs. 18.2 at 12 months) than HIVRR participants.

**Table 3:** Descriptive statistics for outcome variables at baseline and each follow‐up assessment by study arm

In the past 90 days		Baseline (n = 353)	3‐Month (n = 319)	6‐Month (n = 312)	12‐Month (n = 306)
Number of unprotected acts of vaginal and anal intercourse with all partners: Mean (SD)	HIVRR	41.2 (69.8)	11.4 (23.0)	9.6 (18.8)	10.1 (29.0)
	HIVRR + MF	36.6 (68.2)	7.9 (27.3)	9.0 (20.9)	7.0 (17.0)
Number of unprotected acts of vaginal and anal intercourse with paying partners: Mean (SD)	HIVRR	20.3 (37.8)	2.5 (9.8)	1.2 (5.6)	0.4 (2.7)
	HIVRR + MF	20.8 (48.8)	1.5 (10.3)	0.4 (2.1)	0.4 (3.1)
Injected drugs: # (%)	HIVRR	60 (33.7%)	31 (19.1%)	28 (18.0%)	25 (16.5%)
	HIVRR + MF	56 (32.0%)	26 (16.6%)	27 (17.3%)	17 (11.0%)
Unsafe injection: # (%)	HIVRR	59 (33.2%)	31 (19.1%)	25 (16.3%)	24 (15.8%)
	HIVRR + MF	52 (29.7%)	25 (15.9%)	25 (16.0%)	15 (9.7%)
Proportion of income from sex work: mean (SD)	HIVRR	0.57 (0.33)	0.23 (0.31)*	0.17 (0.27)	0.11 (0.24)
	HIVRR + MF	0.58 (0.36)	0.15 (0.25)*	0.13 (0.26)	0.11 (0.27)
Number of sex partners: mean (SD)	HIVRR	19.0 (40.3)	2.4 (5.3)	1.8 (5.4)	0.8 (1.6)
	HIVRR + MF	17.0 (47.7)	2.9 (13.6)	2.2 (14.8)	0.8 (3.5)
Number of paying partners: mean (SD)	HIVRR	13.2 (31.1)	1.3 (3.8)	1.0 (4.7)	0.3 (1.1)
	HIVRR + MF	12.2 (36.4)	1.4 (7.0)	1.5 (14.9)	0.4 (3.3)
Financial self‐efficacy: mean (SD)^a^	HIVRR	12.6 (4.0)	13.0 (4.1)	13.2 (4.4)	14.1 (3.9)
	HIVRR + MF	11.8 (4.0)	13.8 (4.0)	13.9 (4.3)	14.7 (4.1)
Financial safety: mean (SD)^b^	HIVRR	16.5 (3.1)	16.4 (2.8)*	16.6 (3.1)	16.4 (3.2)
	HIVRR + MF	16.4 (3.1)	17.1 (2.4)*	16.6 (3.3)	17.0 (2.6)
Condom use self‐efficacy: mean (SD)^c^	HIVRR	19.7 (6.0)	23.7 (4.5)	23.4 (5.0)	23.0 (5.5)
	HIVRR + MF	20.2 (5.9)	23.9 (3.6)	23.4 (5.0)	23.7 (4.4)
Condom use attitudes: mean (SD)^d^	HIVRR	18.2 (5.2)	20.9 (5.9)*	21.4 (5.6)	22.0 (5.7)
	HIVRR + MF	18.4 (5.5)	22.3 (6.2)*	22.6 (5.9)	22.6 (6.5)
Perceived social support: mean (SD)^e^	HIVRR	44.4 (19.4)	49.9 (20.2)**	51.3 (21.2)	51.8 (23.3)*
	HIVRR + MF	47.8 (19.6)	57.8 (19.4)**	55.7 (22.4)	57.6 (22.3)*
Access to care: mean (SD)^f^	HIVRR	16.4 (3.9)**	17.7 (4.5)**	18.0 (4.3)**	18.2 (4.1)**
	HIVRR + MF	17.8 (4.4)**	19.1 (4.4)**	19.5 (5.0)**	20.5 (4.7)**

HIVRR + MF, HIV risk reduction and microfinance; HIVRR, HIV risk reduction.

^a^ Financial Self‐Efficacy: 4 items, 5‐point Likert scale (1 = “Not at all confident” to 5 = “Extremely confident”)

^b^ financial Safety: 4 items, 5‐point Likert scale (1 = “Very strongly disagree” to 5 = “Very strongly agree”)

^c^ condom Use Self‐Efficacy: 9 items, 3‐point Likert scale (1 = “Not at all confident” to 3 = “Very confident”)

^d^ condom Use Attitudes: 9 items, 5‐point Likert scale (0 = “Strongly disagree” to 4 = “Strongly Agree”)

^e^ Social Support (MSPSS): 12 items, 7‐point Likert scale (1 = “Very strongly disagree” to 7 = “Very strongly agree”)

^f^ Access to Care: 6 items, 5‐point Likert scale (1 = “Strongly disagree”, 5 = “Strongly agree”)

* *p* < 0.05, **p < 0.01 by 2‐tailed *t*‐test or Chi‐squared test at the baseline or each follow‐up assessment

Tables 4 and 5 report multilevel models to estimate quantitatively the effect of change from baseline to each follow‐up assessment and the difference in this change between HIVRR + MF vs. HIVRR on each behavioural outcome measure. Table 4 shows significant differences between arms in the reduction from baseline to the follow‐up assessment in the number of unprotected acts of vaginal and/or anal intercourse with paying partners and the proportion of income from sex work. Participants assigned to HIVRR + MF reported a 72% greater reduction in the number of unprotected acts of vaginal and/or anal intercourse with paying partners at the 6‐month assessment (IRR = 0.28, 95% CI = 0.08, 0.92) and a 10% greater reduction in the portion of income from sex work at the 3‐month assessment (b = −0.10, 95% CI = −0.17, −0.02) than participants in the HIVRR arm. In addition, results show significant reductions in primary behavioural outcomes for both arms at each follow‐up compared to baseline. For example HIVRR participants reported an 82% reduction at three months (IRR = 0.18, 95% CI = 0.10, 0.31), an 85% reduction at six months (IRR = 0.15, 95% CI = 0.08, 0.26), and a 90% reduction at 12 months (IRR = 0.10, 95% CI = 0.05, 0.19) in the number of acts of unprotected vaginal and/or anal intercourse with all partners, compared to their baseline assessment. Likewise, participants assigned to HIVRR + MF reported a 92% reduction at three months (IRR = 0.08, 95% CI = 0.04, 0.16), 88% reduction at six months (IRR = 0.12, 95% CI = 0.07, 0.23), and a 91% reduction at 12 months (IRR = 0.09, 95% CI = 0.04, 0.16) in the number of acts of unprotected vaginal and/or anal intercourse with all partners, compared to their baseline assessment.

**Table 4:** Random effects models for primary behavioural outcomes: change from the baseline to each follow‐up assessment by study arm

In the past 90 days		Change from baseline			
		Entire follow‐up	3‐month	6‐month	12‐month
Number of unprotected acts of vaginal and anal intercourse with all partners (IRR)	HIVRR	0.15 [0.09, 0.25]** (*p* < 0.001)	0.18 [0.10, 0.31]** (*p* < 0.001)	0.15 [0.08, 0.26]** (*p* < 0.001)	0.10 [0.05, 0.19]** (*p* < 0.001)
	HIVRR + MF	0.11 [0.06, 0.18]** (*p* < 0.001)	0.08 [0.04, 0.16]** (*p* < 0.001)	0.12 [0.07, 0.23]** (*p* < 0.001)	0.09 [0.04, 0.16]** (*p* < 0.001)
	HIVRR vs. HIVRR + MF	0.70 [0.37, 1.33] (*p* = 0.277)	0.48 [0.22, 1.07] (*p* = 0.071)	0.86 [0.39, 1.90] (*p* = 0.702)	0.87 [0.38, 2.00] (*p* = 0.749)
Number of unprotected acts of vaginal and anal intercourse with paying partners (IRR)	HIVRR	0.01 [0.005, 0.02]** (*p* < 0.001)	0.02 [0.01, 0.05]** (*p* < 0.001)	0.01 [0.004, 0.02]** (*p* < 0.001)	0.002 [0.001, 0.004]** (*p* < 0.001)
	HIVRR + MF	0.004 [0.002, 0.01]** (*p* < 0.001)	0.01 [0.003, 0.02]** (*p* < 0.001)	0.003 [0.001, 0.01]** (*p* < 0.001)	0.002 [0.001, 0.01]** (*p* < 0.001)
	HIVRR vs. HIVRR + MF	0.40 [0.16, 0.98]* (*p* = 0.046)	0.36 [0.12, 1.07] (*p* = 0.066)	0.28 [0.08, 0.92]* (*p* = 0.035)	1.14 [0.29, 4.50] (*p* = 0.851)
Injected drugs (OR)	HIVRR	0.10 [0.05, 0.22]** (*p* < 0.001)	0.13 [0.05, 0.31]** (*p* < 0.001)	0.11 [0.04, 0.28]** (*p* < 0.001)	0.07 [0.03, 0.20]** (*p* < 0.001)
	HIVRR + MF	0.10 [0.05, 0.20]** (*p* < 0.001)	0.11 [0.04, 0.27]** (*p* < 0.001)	0.14 [0.06, 0.35]** (*p* < 0.001)	0.05 [0.02, 0.13]** (*p* < 0.001)
	HIVRR vs. HIVRR + MF	0.93 [0.34, 2.55] (*p* = 0.885)	0.85 [0.24, 2.97] (*p* = 0.803)	1.31 [0.37, 4.59] (*p* = 0.675)	0.63 [0.16, 2.51] (*p* = 0.517)
Unsafe injection (OR)	HIVRR	0.09 [0.04, 0.20]** (*p* < 0.001)	0.13 [0.05, 0.32]** (*p* < 0.001)	0.08 [0.03, 0.21]** (*p* < 0.001)	0.06 [0.02, 0.18]** (*p* < 0.001)
	HIVRR + MF	0.10 [0.05, 0.22]** (*p* < 0.001)	0.12 [0.05, 0.32]** (*p* < 0.001)	0.15 [0.06, 0.37]** (*p* < 0.001)	0.05 [0.02, 0.13]** (*p* < 0.001)
	HIVRR vs. HIVRR + MF	1.16 [0.41, 3.28] (*p* = 0.784)	0.99 [0.28, 3.51] (*p* = 0.983)	1.97 [0.53, 7.33] (*p* = 0.311)	0.73 [0.18, 3.00] (*p* = 0.659)
Proportion of income from sex work (b)	HIVRR	−0.40 [−0.44, −0.35]** (*p* < 0.001)	−0.34 [−0.39, −0.29]** (*p* < 0.001)	−0.40 [−0.45, −0.34]** (*p* < 0.001)	−0.46 [−0.51, −0.40]** (*p* < 0.001)
	HIVRR + MF	−0.45 [−0.49, −0.40]** (*p* < 0.001)	−0.44 [−0.49, −0.38]** (*p* < 0.001)	−0.45 [−0.50, −0.39]** (*p* < 0.001)	–0.46 [−0.52, −0.41]** (*p* < 0.001)
	HIVRR vs. HIVRR + MF	−0.05 [−0.11, 0.01] (*p* = 0.114)	–0.10 [−0.17, −0.02]* (*p* = 0.013)	−0.05 [−0.12, 0.03] (*p* = 0.232)	−0.003 [−0.08, 0.07] (*p* = 0.935)

Covariate adjustments are age, education, marital status, homelessness and food insecurity and site. HIVRR, HIV risk reduction; HIVRR + MF, HIV risk reduction and Microfinance.

* *p* < 0.05

** *p* < 0.01.

**Table 5:** Random effects models for secondary behavioural outcomes: change from the baseline to each follow‐up assessment by study arm

	Change from baseline			
	Entire follow‐up	3‐Month	6‐Month	12‐Month
Number of sex partners (IRR)				
HIVRR	0.09 [0.07, 0.11]** (*p* < 0.001)	0.13 [0.09, 0.17]** (*p* < 0.001)	0.09 [0.06, 0.12]** (*p* < 0.001)	0.05 [0.03, 0.07]** (*p* < 0.001)
HIVRR + MF	0.08 [0.06, 0.11]** (*p* < 0.001)	0.11 [0.08, 0.15]** (*p* < 0.001)	0.09 [0.06, 0.12]** (*p* < 0.001)	0.05 [0.04, 0.08]** (*p* < 0.001)
HIVRR vs. HIVRR + MF	0.95 [0.66, 1.35] (*p* = 0.757)	0.86 [0.56, 1.33] (*p* = 0.499)	1.01 [0.64, 1.59] (*p* = 0.969)	1.12 [0.68, 1.83] (*p* = 0.662)
Number of paying partners (IRR)				
HIVRR	0.03 [0.02, 0.05]** (*p* < 0.001)	0.06 [0.04, 0.09]** (*P* < 0.001)	0.03 [0.02, 0.06]** (*p* < 0.001)	0.01 [0.01, 0.02]** (*p* < 0.001)
HIVRR + MF	0.03 [0.02, 0.04]** (*p* < 0.001)	0.05 [0.03, 0.08]** (*p* < 0.001)	0.03 [0.01, 0.04]** (*p* < 0.001)	0.01 [0.01, 0.02]** (*p* < 0.001)
HIVRR vs. HIVRR + MF	0.83 [0.48, 1.43] (*p* = 0.502)	0.89 [0.46, 1.70] (*p* = 0.718)	0.76 [0.37, 1.55] (*p* = 0.446)	0.93 [0.39, 2.22] (*p* = 0.874)
Financial self‐efficacy (b)				
HIVRR	0.78 [0.23, 1.33]** (*p* = 0.006)	0.35 [−0.33, 1.02] (*p* = 0.312)	0.55 [−0.13, 1.23] (*p* = 0.115)	1.48 [0.79, 2.17]** (*p* < 0.001)
HIVRR + MF	2.24 [1.69, 2.80]** (*p* < 0.001)	1.94 [1.26, 2.62]** (*p* < 0.001)	2.02 [1.34, 2.70]** (*p* < 0.001)	2.79 [2.10, 3.47]** (*p* < 0.001)
HIVRR vs. HIVRR + MF	1.47 [0.68, 2.25]** (*p* < 0.001)	1.59 [0.63, 2.55]** (*p* = 0.001)	1.47 [0.50, 2.43]** (*p* = 0.003)	1.31 [0.33, 2.28]** (*p* = 0.008)
Financial safety (b)				
HIVRR	−0.003 [−0.43, 0.42] (*p* = 0.987)	−0.06 [−0.58, 0.45] (*p* = 0.806)	0.09 [−0.44, 0.61] (*p* = 0.742)	−0.03 [−0.56, 0.50] (*p* = 0.908)
HIVRR + MF	0.45 [0.02, 0.87]* (*p* = 0.040)	0.62 [0.10, 1.14]* (*p* = 0.020)	0.16 [−0.37, 0.68] (*p* = 0.558)	0.56 [0.03, 1.08]* (*p* = 0.038)
HIVRR vs. HIVRR + MF	0.45 [−0.15, 1.05] (*p* = 0.142)	0.69 [−0.05, 1.42] (*p* = 0.068)	0.07 [−0.67, 0.81] (*p* = 0.856)	0.59 [−0.16, 1.34] (*p* = 0.122)
Condom use self‐efficacy (b)				
HIVRR	3.67 [2.97, 4.38]** (*p* < 0.001)	3.98 [3.11, 4.84]** (*p* < 0.001)	3.77 [2.89, 4.64]** (*p* < 0.001)	3.24 [2.36, 4.12]** (*p* < 0.001)
HIVRR + MF	3.48 [2.77, 4.19]** (*p* < 0.001)	3.73 [2.85, 4.60]** (*p* < 0.001)	3.22 [2.34, 4.10]** (*p* < 0.001)	3.49 [2.61, 4.37]** (*p* < 0.001)
HIVRR vs. HIVRR + MF	−0.19 [−1.19, 0.81] (*p* = 0.708)	−0.25 [−1.48, 0.98] (*p* = 0.689)	−0.55 [−1.79, 0.69] (*p* = 0.385)	0.25 [−0.99, 1.50] (*p* = 0.690)
Condom use attitude (b)				
HIVRR	3.18 [2.40, 3.96]** (*p* < 0.001)	2.67 [1.71, 3.63]** (*p* < 0.001)	3.16 [2.19, 4.13]** (*p* < 0.001)	3.77 [2.79, 4.74]** (*p* < 0.001)
HIVRR + MF	3.98 [3.19, 4.76]** (*p* < 0.001)	3.86 [2.89, 4.82]** (*p* < 0.001)	4.01 [3.04, 4.98]** (*p* < 0.001)	4.07 [3.10, 5.05]** (*p* < 0.001)
HIVRR vs. HIVRR + MF	0.79 [−0.31, 1.90] (*p* = 0.159)	1.19 [−0.17, 2.55] (*p* = 0.087)	0.85 [−0.52, 2.22] (*p* = 0.225)	0.31 [−1.07, 1.68] (*p* = 0.664)
Perceived social support (b)				
HIVRR	6.35 [3.33, 9.38]** (*p* < 0.001)	5.31 [1.61, 9.02]** (*p* = 0.005)	6.88 [3.12, 10.63]** (*p* < 0.001)	6.95 [3.17, 10.73]** (*p* < 0.001)
HIVRR + MF	8.76 [5.73, 11.79]** (*p* < 0.001)	9.48 [5.73, 13.22]** (*p* < 0.001)	7.55 [3.79, 11.30]** (*p* < 0.001)	9.26 [5.49, 13.02]** (*p* < 0.001)
HIVRR vs. HIVRR + MF	2.40 [−1.87, 6.68] (*p* = 0.271)	4.16 [−1.11, 9.43] (*p* = 0.122)	0.67 [−4.64, 5.98] (*p* = 0.805)	2.31 [−3.03, 7.64] (*p* = 0.397)
Access to care (b)				
HIVRR	1.50 [0.93, 2.06]** (*p* < 0.001)	1.30 [0.61, 1.99]** (*p* < 0.001)	1.45 [0.75, 2.15]** (*p* < 0.001)	1.76 [1.06, 2.47]** (*p* < 0.001)
HIVRR + MF	1.66 [1.09, 2.23]** (*p* < 0.001)	1.03 [0.33, 1.73]** (*p* = 0.004)	1.53 [0.83, 2.23]** (*p* < 0.001)	2.45 [1.75, 3.15]** (*p* < 0.001)
HIVRR vs. HIVRR + MF	0.17 [−0.64, 0.97] (*p* = 0.686)	−0.27 [−1.25, 0.71] (*p* = 0.589)	0.08 [−0.91, 1.07] (*p* = 0.870)	0.68 [−0.31, 1.68] (*p* = 0.177)

Covariate adjustments are age, education, marital status, homelessness and food insecurity and site. HIVRR, HIV Risk Reduction; HIVRR + MF, HIV Risk Reduction & Microfinance.

* *p* < 0.05

** *p* < 0.01.

Table 5 presents the findings of multilevel models for secondary behavioural outcome measures. The findings show participants assigned to HIVRR + MF had significantly greater increase in financial self‐efficacy at each follow‐up (b = 1.59, 95% CI = 0.63, 2.55 at three months; b = 1.47, 95% CI = 0.50, 2.43 at six months; and b = 1.31, 95% CI = 0.33, 2.28 at 12‐months) than participants in the HIVRR arm. Although there were no significant differences between arms for other secondary behavioural outcomes, participants in both arms reported significant reductions in the numbers of all sex partners and paying partners, and significant increases in financial self‐efficacy, condom use self‐efficacy, condom use attitude, perceived social support, and access to care at the follow‐up compared to baseline assessment. Only HIVRR + MF participants reported significant increases in financial safety at the follow‐up compared to baseline assessment.

## 4 DISCUSSION

The results show that both HIVRR control and HIVRR + MF treatment participants significantly reduced sexual and drug use risk behaviours at post‐intervention assessments compared to baseline, including numbers of commercial partners, unprotected sex acts with all partners, and unsafe injections. Both arms also showed improvements in financial outcomes, including the proportion of their income from sex work, financial self‐efficacy and safety, as well as on condom use attitudes and self‐efficacy, social support, and self‐reported access to medical care. Neither arm showed significant decreases in HIV or STI incidence; this lack of support for biological STI outcomes is consistent with other evidence‐based interventions [23, 24].

Unlike other studies to‐date there were very few differences between the two study arms [12, 13, 14, 15]. The HIVRR + MF arm showed a significantly greater reduction in the number of unprotected sex acts with paying partners than the HIVRR control arm at the 6‐month follow up compared to the baseline, but this difference was not sustained over the full follow‐up period. Likewise, HIVRR + MF participants showed 10% greater reduction in the proportion of income from sex work than HIVRR participants at the 3‐month assessment, but this difference was not sustained at other follow‐up points. Only on financial self‐efficacy did the HIVRR + MF arm show significantly greater improved performance over the HIVRR arm. Therefore it seems that the extra microfinance components received by the HIVRR + MF treatment arm did not provide any measurable significant additional protection beyond the HIVRR alone.

There are several explanations for the lack of significant difference between study arms on most risk outcomes. First, we consider whether the HIVRR intervention provided to both arms was itself adequate in reducing risk behaviours. This robust intervention had been carefully adapted and tailored to the needs of FSW who use drugs in Kazakhstan through a multi‐step process, and both arms showed high retention, participation, and engagement [17]. The high prevalence of STIs, homelessness, and food insecurity at baseline suggests that participants in both arms had diverse and acute unmet needs prior to the intervention. Indeed, both arms reported significantly increased access to care at all follow‐ups, suggesting that HIVRR alone removed many of the barriers to medical and social services that drive these risks. Furthermore, participation in a cohort may have enhanced peer connections and support in both arms, which may facilitate linkage to community resources and reduction in risk behaviours [25, 26, 27]. The increases in perceived social support for both arms necessitate further exploration of the impact of FSW peer networks on risk reduction.

Another explanation is that the MF intervention components were not sufficient to overcome barriers to successful transition to alternative employment. These may include environmental barriers such as employment or job availability, economic barriers such as the higher relative income from sex work compared to alternative jobs, or persistent interpersonal barriers such as stigma or drug use, impeding perceived or real access to employment [28]. As noted elsewhere [17], Kazakhstan underwent a recession in 2015 and 2016 that may have impacted participants’ abilities to save money and to find sustainable employment. Another barrier may be the length of time needed to successfully transition to alternative employment.

These unsustained, yet significant differences between arms suggest the need to assess the differential impacts of various microfinance components. Others have highlighted the importance of disentangling impacts of combination interventions prior to further scale‐up [29]. Additionally, we consider whether additional employment‐related environmental supports could enhance the microfinance components. For example future microfinance programmes including vocational training elements must work closely with NGOs and employment agencies to ensure alternative employment opportunities exist for trained participants.

### 4.1 Strengths and limitations

Our study utilized a convenience sample of FSW who used drugs recruited from specific venues and through peer networks in two cities. This limits the generalizability of our findings to FSW populations in other settings, both in Central Asia and globally. There is also potential of participant response bias, including social pressures to under‐report numbers of sexual partners and risk behaviours. We mitigated this risk by utilizing a private computer‐assisted self‐interview process. Finally, because we utilized peer referral recruitment, participants may have had friends or acquaintances in different cohorts, and therefore contamination between study arms is possible. However, given the complexity of MF intervention components (e.g. specialized vocational skills), we do not believe this had a large impact on outcomes.

## 5 CONCLUSIONS

Findings suggest that there may be structural limitations to the promise of microfinance for HIV risk reduction among FSW who use drugs. Nova is one of the first studies among this vulnerable and under‐researched population, and its acceptability and feasibility, described elsewhere [17], are promising. However, further adaptation of the microfinance elements, as well as broader policy changes that can expand employment opportunities may be necessary to observe the sustainable improved impact of microfinance. Given accumulated knowledge regarding the structural barriers to risk reduction, particularly in low and middle‐income countries and among this vulnerable population, we encourage continued examination of the potential of microfinance for HIV risk reduction.

## COMPETING INTERESTS

The authors declare that they have no competing interests.

## AUTHORS’ CONTRIBUTIONS

NE and SSW are joint principal investigators (PIs) and guided overall conceptual design for the study and intervention and preparation of the manuscript. AT and SP contributed to the conceptual design, and oversaw study implementation in the field. MC conducted the data analysis and drafted the data analysis section of this manuscript. TM and GM were project directors and drafted and implemented study protocols, measures and data management. TM oversaw drafting and revising of this manuscript. AK contributed to protocol design and implemented study protocols in the field sites. BB, AD and KK advised on study protocols and facilitated participant biotesting and treatment procedures in field sites. All authors read and approved this manuscript.

## References

[1] Baral S , Beyrer C , Muessig K , Poteat T , Wirtz AL , Decker MR , et al. Burden of HIV among female sex workers in low‐income and middle‐income countries: a systematic review and meta‐analysis. Lancet Infect Dis. 2012;12(7):538–49. 22424777 10.1016/S1473-3099(12)70066-X

[2] Baral S , Todd CS , Aumakhan B , Lloyd J , Delegchoimbol A , Sabin K . HIV among female sex workers in the Central Asian Republics, Afghanistan, and Mongolia: contexts and convergence with drug use. Drug Alcohol Depend. 2013;132 Suppl 1:S13–6. 23954072 10.1016/j.drugalcdep.2013.07.004

[3] Kazakh Scientific Center for Dermatology and Infectious Diseases . HIV surveillance data. Kazakhstan: Almaty; 2020.

[4] El‐Bassel N , Wechsberg WM , Shaw SA . Dual HIV risk and vulnerabilities among women who use or inject drugs: no single prevention strategy is the answer. Curr Opin HIV AIDS. 2012;7(4):326–31. 22498480 10.1097/COH.0b013e3283536ab2 PMC6330012

[5] El‐Bassel N , Strathdee SA . Women who use or inject drugs: an action agenda for women‐specific, multilevel, and combination HIV prevention and research. J Acquir Immune Defic Syndr. 2015;69 Suppl 2:S182–90. 25978486 10.1097/QAI.0000000000000628 PMC4932853

[6] Shannon K , Strathdee SA , Goldenberg SM , Duff P , Mwangi P , Rusakova M , et al. Global epidemiology of HIV among female sex workers: influence of structural determinants. Lancet. 2015;385(9962):55–71. 25059947 10.1016/S0140-6736(14)60931-4 PMC4297548

[7] Strathdee SA , West BS , Reed E , Moazen B , Azim T , Dolan K . Substance use and HIV among female sex workers and female prisoners: risk environments and implications for prevention, treatment, and policies. J Acquir Immune Defic Syndr. 2015;69 Suppl 2:S110–7. 25978477 10.1097/QAI.0000000000000624 PMC4493865

[8] Dworkin SL , Blankenship K . Microfinance and HIV/AIDS prevention: assessing its promise and limitations. AIDS Behav. 2009;13(3):462–9. 19294500 10.1007/s10461-009-9532-3 PMC3770268

[9] Arrivillaga M , Salcedo JP . A systematic review of microfinance‐based interventions for HIV/AIDS prevention. AIDS Educ Prev. 2014;26(1):13–27. 24450275 10.1521/aeap.2014.26.1.13

[10] Kennedy CE , Fonner VA , O'Reilly KR , Sweat MD . A systematic review of income generation interventions, including microfinance and vocational skills training, for HIV prevention. AIDS Care. 2014;26(6):659–73. 24107189 10.1080/09540121.2013.845287 PMC3943565

[11] Cui RR , Lee R , Thirumurthy H , Muessig KE , Tucker JD . Microenterprise development interventions for sexual risk reduction: a systematic review. AIDS Behav. 2013;17(9):2864–77. 23963497 10.1007/s10461-013-0582-1 PMC3877769

[12] Sherman SG , German D , Cheng Y , Marks M , Bailey‐Kloche M . The evaluation of the JEWEL project: an innovative economic enhancement and HIV prevention intervention study targeting drug using women involved in prostitution. AIDS Care. 2006;18(1):1–11. 16282070 10.1080/09540120500101625

[13] Sherman SG , Srikrishnan AK , Rivett KA , Liu SH , Solomon S , Celentano DD . Acceptability of a microenterprise intervention among female sex workers in Chennai. India. AIDS Behav. 2010;14(3):649–57. 20352320 10.1007/s10461-010-9686-z

[14] Odek WO , Busza J , Morris CN , Cleland J , Ngugi EN , Ferguson AG . Effects of micro‐enterprise services on HIV risk behaviour among female sex workers in Kenya's urban slums. AIDS Behav. 2009;13(3):449–61. 18998204 10.1007/s10461-008-9485-y

[15] Witte SS , Aira T , Tsai LC , Riedel M , Offringa R , Chang M , et al. Efficacy of a savings‐led microfinance intervention to reduce sexual risk for HIV among women engaged in sex work: a randomized clinical trial. Am J Public Health. 2015;105(3):e95–102. 10.2105/AJPH.2014.302291 PMC4330842 25602889

[16] McCrimmon T , Witte S , Mergenova G , Terlikbayeva A , Primbetova S , Kuskulov A , et al. Microfinance for women at high risk for HIV in Kazakhstan: study protocol for a cluster‐randomized controlled trial. Trials. 2018;19(1):187. 29558982 10.1186/s13063-018-2566-y PMC5859522

[17] Mergenova G , El‐Bassel N , McCrimmon T , Terlikbayeva A , Primbetova S , Riedel M , et al. Project nova: a combination hiv prevention and microfinance intervention for women who engage in sex work and use drugs in Kazakhstan. AIDS Behav. 2019;23(1):1–14. 10.1007/s10461-018-2268-1 PMC6790132 30194502

[18] National Institute on Drug Abuse . Risk behavior assessment. Rockville, MD: National Institute on Drug Abuse (Community Research Branch); 1993.

[19] Zimet GD , Dahlem NW , Zimet SG , Farley GK . The Multidimensional scale of perceived social support. J Pers Assess. 1988;52(1):30–41. 10.1080/00223891.1990.9674095 2280326

[20] Opportunities M . Global financial education program. Washington, DC: Microfinance Opportunities; 2002.

[21] Weaver TL , Sanders CK , Campbell CL , Schnabel M . Development and preliminary psychometric evaluation of the domestic violence–related financial issues scale (DV‐FI). J Interpers Violence. 2009;24(4):569–85. 18445829 10.1177/0886260508317176

[22] El‐Bassel N , Gilbert L , Terlikbayeva A , Beyrer C , Wu E , Chang M , et al. Effects of a couple‐based intervention to reduce risks for HIV, HCV, and STIs among drug‐involved heterosexual couples in Kazakhstan: a randomized controlled trial. J Acquir Immune Defic Syndr. 2014;67(2):196–203. 24991973 10.1097/QAI.0000000000000277 PMC4162759

[23] Crepaz N , Tungol‐Ashmon MV , Higa DH , Vosburgh W , Mullins MM , Barham T , et al. A systematic review of interventions for reducing HIV risk behaviors among people living with HIV in the United States, 1988–2012. AIDS. 2014;28(5):633–56. 24983541 10.1097/QAD.0000000000000108 PMC4678954

[24] El‐Bassel N , Gilbert L , Goddard‐Eckrich D , Chang M , Wu E , Goodwin S , et al. Effectiveness of a couple‐based HIV and sexually transmitted infection prevention intervention for men in community supervision programs and their female sexual partners: a randomized clinical trial. JAMA Network Open. 2019;2:e191139. 30924895 10.1001/jamanetworkopen.2019.1139 PMC6450427

[25] Campbell C . Selling sex in the time of AIDS: the psycho‐social context of condom use by sex workers on a Southern African mine. Soc Sci Med. 2000;50(4):479–94. 10641801 10.1016/s0277-9536(99)00317-2

[26] Rodríguez DC , Krishnan AK , Kumarasamy N , Krishnan G , Solomon D , Johnson S , et al. Two sides of the same story: alcohol use and HIV risk taking in South India. AIDS Behav. 2010;14 Suppl 1:136–46. 10.1007/s10461-010-9722-z PMC2900584 20544382

[27] Tsai LC , Carlson CE , Aira T , Norcini Pala A , Riedel M , Witte SS . The impact of a microsavings intervention on reducing violence against women engaged in sex work: a randomized controlled study. BMC Int Health Hum Rights. 2016;16(1):27. 27793147 10.1186/s12914-016-0101-3 PMC5086041

[28] Stringer KL , Marotta P , Baker E , Turan B , Kempf MC , Drentea P , et al. Substance use stigma and antiretroviral therapy adherence among a drug‐using population living with HIV. AIDS Patient Care STDS. 2019;33(6):282–93. 31166784 10.1089/apc.2018.0311 PMC6588110

[29] Peterman A , Palermo TM , Ferrari G . Still a leap of faith: microfinance initiatives for reduction of violence against women and children in low‐income and middle‐income countries. BMJ Global Health. 2018;3:e001143. 10.1136/bmjgh-2018-001143 PMC6278915 30588347

